# Antioxidant, Antitumoral, Antimicrobial, and Prebiotic Activity of Magnetite Nanoparticles Loaded with Bee Pollen/Bee Bread Extracts and 5-Fluorouracil

**DOI:** 10.3390/antiox13080895

**Published:** 2024-07-24

**Authors:** Cornelia-Ioana Ilie, Angela Spoiala, Cristina Chircov, Georgiana Dolete, Ovidiu-Cristian Oprea, Bogdan-Stefan Vasile, Simona Adriana Crainiceanu, Adrian-Ionut Nicoara, Ioana Cristina Marinas, Miruna Silvia Stan, Lia-Mara Ditu, Anton Ficai, Eliza Oprea

**Affiliations:** 1 Department of Science and Engineering of Oxide Materials and Nanomaterials, Faculty of Chemical Engineering and Biotechnologies, National University of Science and Technology Politehnica Bucharest, 011061 Bucharest, Romania; cornelia_ioana.ilie@upb.ro (C.-I.I.); angela.spoiala@upb.ro (A.S.); cristina.chircov@upb.ro (C.C.); georgiana.dolete@upb.ro (G.D.); adriana.crainiceanu@upb.ro (S.A.C.); adrian.nicoara@upb.ro (A.-I.N.); 2 National Centre for Micro and Nanomaterials and National Centre for Food Safety, National University of Science and Technology Politehnica Bucharest, 060042 Bucharest, Romania; bogdan.vasile@upb.ro; 3 Research Center for Advanced Materials, Products and Processes, National University of Science and Technology Politehnica Bucharest, 060042 Bucharest, Romania; 4 Academy of Romanian Scientists, 010719 Bucharest, Romania; ovidiu.oprea@upb.ro; 5 Department of Inorganic Chemistry, Physical Chemistry and Electrochemistry, Faculty of Chemical Engineering and Biotechnologies, National University of Science and Technology Politehnica Bucharest, 011061 Bucharest, Romania; 6 The Research Institute, University of Bucharest, 050663 Bucharest, Romaniamiruna.stan@bio.unibuc.ro (M.S.S.); 7 Department of Biochemistry, Faculty of Biology, University of Bucharest, 050095 Bucharest, Romania; 8 Department of Botany and Microbiology, Faculty of Biology, University of Bucharest, 060101 Bucharest, Romania; eliza.oprea@g.unibuc.ro

**Keywords:** bee bread, bee pollen, antioxidant, antitumoral and antibacterial agents, drug delivery systems

## Abstract

The gut microbiota dysbiosis that often occurs in cancer therapy requires more efficient treatment options to be developed. In this concern, the present research approach is to develop drug delivery systems based on magnetite nanoparticles (MNPs) as nanocarriers for bioactive compounds. First, MNPs were synthesized through the spraying-assisted coprecipitation method, followed by loading bee pollen or bee bread extracts and an antitumoral drug (5-fluorouracil/5-FU). The loaded-MNPs were morphologically and structurally characterized through transmission electron microscopy (TEM), selected area electron diffraction (SAED), scanning electron microscopy (SEM), X-ray diffraction (XRD), Fourier transform infrared spectroscopy (FT-IR), Dynamic Light Scattering (DLS), and thermogravimetric analysis. UV-Vis spectroscopy was applied to establish the release profiles and antioxidant activity. Furthermore, the antibacterial and antitumoral activity of loaded-MNPs was assessed. The results demonstrate that MNPs with antioxidant, antibacterial, antiproliferative, and prebiotic properties are obtained. Moreover, the data highlight the improvement of 5-FU antibacterial activity by loading on the MNPs’ surface and the synergistic effects between the anticancer drug and phenolic compounds (PCs). In addition, the prolonged release behavior of PCs for many hours (70–75 h) after the release of 5-FU from the developed nanocarriers is an advantage, at least from the point of view of the antioxidant activity of PCs. Considering the enhancement of *L. rhamnosus* MF9 growth and antitumoral activity, this study developed promising drug delivery alternatives for colorectal cancer therapy.

## 1. Introduction

Due to its unique structural, electrical, and magnetic properties, metal oxide nanoparticles such as magnetite nanoparticles (MNPs) are given tremendous attention. Over the past few decades, researchers and scientists have studied their applications in memory devices, data storage, water purification, bioprocessing, drug delivery, hyperthermia, magnetic resonance imaging, biosensors, electronic devices, aerospace, etc. [1,2].

Considering various nanoparticle synthesis approaches, chemical coprecipitation is the most used technique for synthesizing MNPs, mainly due to its eco-friendly precursors, cost-effectiveness, and easy procedure [3].

MNPs are used in several biomedical applications, especially for antimicrobial and anticancer therapies [4,5,6,7]. Also, due to their properties like precise targeting, low toxicity, biocompatibility, nanometric size, etc., MNPs can be internalized and guided with a magnetic field to the tissue/organ of interest [8,9]. Furthermore, MNPs can be functionalized/loaded with bioactive compounds (like antineoplastic drugs, antibiotics (ATBs), PCs, natural extracts, etc.) to develop multi-target drug delivery systems (DDS) [10,11,12,13,14,15,16].

Colorectal cancer (CRC) is a type of malignancy that affects the colon or rectum and, according to the World Health Organization, is the third most frequent cancer worldwide. Mainly, CRC affects older individuals and is the second leading cause of mortality rates, with the highest incidence rate in Australia and Europe [17,18]. It is estimated that in 2040, there will be an increase of 3.2 million new cases and 1.6 million deaths compared to 2020, when approximately 1.9 million new CRC cases and 930,000 deaths were reported [18]. However, gut microbiota dysbiosis may also play a key risk factor in promoting CRC by enhancing bacterial populations that stimulate tumorigenesis/exaggerated immune responses [19,20,21,22]. Recent studies suggest a strong correlation between gut microbiota imbalance and mechanisms of colorectal tumorigenesis. Moreover, since patients with CRC present a richness of procarcinogenic taxa (*Fusobacterium* sp., *Bacteroides* sp., *Porphyromonas* sp., and *Escherichia coli)* and less protective taxa, like *Roseburia* sp., gut microbiota may represent an important biomarker [21,23,24,25]. 

CRC treatments, such as radiotherapy and chemotherapy, can kill the tumor cells and significantly induce damage to the gut microbiota balance [26]. The related antineoplastic agents for CRC treatment are 5-fluorouracil (5-FU), oxaliplatin, irinotecan, capecitabine, cetuximab, etc., which generate dysbiosis, like diarrhea and intestinal mucositis [26,27,28]. Likewise, the gut microbiota composition is highly linked to the efficacity of antitumoral agents, and in general, during treatment, patients develop severe drug-resistant infections [29]. Additionally, some ATBs like ceftazidime, cefepime, vancomycin, imipenem, neomycin, and metronidazole diminished the 5-FU efficacity in CRC treatment [30,31]. Furthermore, recent studies have shown that exposure to ATB induces gut dysbiosis that is highly linked to CRC [32,33,34]. Consequently, current research approaches to developing multi-target systems could potentiate the effectiveness of chemotherapeutics and diminish the adverse effects by combining them with phytochemicals [35]. 

The adverse effects of antineoplastic agents on the gut can be reduced by using probiotics, prebiotic compounds, antioxidants, symbiotics, and postbiotics as adjuvants in cancer treatment [36]. PCs can represent a prebiotic substrate due to their resistance to host digestion, ability to be metabolized by microorganisms, and stimulation of the probiotic bacteria. Also, they possess antimicrobial, antioxidant, and antitumoral properties [37,38,39,40]. Additionally, other phytochemicals, such as carotenoids, organosulfur compounds, alkaloids, etc., present chemopreventive and antitumoral properties [41]. An excellent source rich in prebiotic compounds can be bee pollen (BP) and bee bread (BB), which can significantly stimulate the growth of probiotic bacteria and thus be considered a source of probiotics [42,43,44,45,46,47].

Antioxidants may act by reducing oxidative stress, modulating inflammation, and improving gut function to alleviate the adverse effects of chemotherapy on the gut microbiota. Some evidence suggests that using antioxidants may help reduce the adverse effects of chemotherapy on the gut microbiota in CRC [48].

Clinical trials in CRC patients have suggested that antioxidants [49,50,51], probiotics [52], and prebiotic supplements [53,54] may reduce the severity of chemotherapy-induced adverse effects and improve gut microbial diversity.

This study presents the development of magnetite-based drug delivery nanocarriers to improve the antitumoral activity of 5-FU and alleviate the adverse effects against gut microbiota. The MNPs were synthesized through a spray-assisted method and loaded with BP/BB extracts and 5-FU. The BPEs and BBEs were characterized in previous studies [42,43]. First, the loaded-MNPs were morphologically and structurally characterized through physical analysis. The bioactive agents’ release behavior was evaluated for 5-FU and PCs, and their antioxidant activity was assessed through three assays. The aim of this research is based on using small concentrations of extracts and 5-FU in order to establish the influence of each bioactive compound and the effects against a selected bacteria with probiotic potential. Furthermore, this study’s novelty involves enhancing the inhibitory activity of bacterial adhesion capacity to the inert substratum induced by the synthesized nanocarriers. 

## 2. Materials and Methods

### 2.1. Materials

Iron chloride (97% reagent grade), sodium hydroxide (≥98%), silver nitrate (≥99.5% purity), 5-FU (≥99.9%, HPLC grade), Folin–Ciocalteu reagent, gallic acid (≥98.0%), sodium carbonate (≥99.5%), 2,2-diphenyl-1-picrylhydrazyl (DPPH), FRAP reagent, CuCl_2_, neocuproin, Trolox (6-hydroxy-2,5,7,8-tetramethylchroman-2-carboxylic acid), ethanol (≥99.8%), methanol, Nutrient Broth No. 2 (NB), Sabouraud Glucose Agar (Sab) with chloramphenicol, Man-Rogosa-Sharpe (MRS), agar, acetic acid (AcA), and crystal violet (CV), were purchased from Sigma-Aldrich (Darmstadt, Germany). Ammonium iron sulfate crystallized hexahydrate with ≥98% purity was acquired from Roth (Karlsruhe, Germany), and the anhydrous trisodium citrate (99% purity) was from Alfa Aesar (Ward Hill, MA, USA). All chemicals were used without further purification. All strains tested in this paper were provided from the Microorganisms Collection of the Department of Microbiology, Faculty of Biology of the University of Bucharest. 

### 2.2. Synthesis of Magnetic Nanoparticles and Loading with Bioactive Compounds

**Figure 1:**
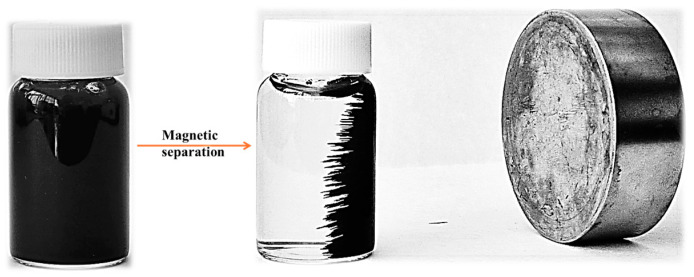
Fe_3_O_4_@citrate magnetic NPs suspension in water and the magnetic attraction.

The Fe_3_O_4_ NPs stabilized with anhydrous trisodium citrate were synthesized by the spraying-assisted coprecipitation method using ammonium iron sulfate hexahydrate and iron chloride as precursors [55,56]. The obtained Fe_3_O_4_@citrate magnetic NPs (Figure 1) were dried and characterized to ensure that the obtained NPs presented the desired properties. After that, 1 g of the previously obtained Fe_3_O_4_@citrate magnetic NPs were loaded with 10 mL hydroethanolic bee pollen/bee bread extracts (BPEs/BBEs) by grinding until the solvent was evaporated. The BPEs and BBEs loaded on the surface of MNPs were previously characterized [42,43], and the samples with the highest concentration of PCs were used in this study. The anticancer drug (5-FU) was first solubilized in ethanol and loaded on the surface of the Fe_3_O_4_@BPEs/BBEs following the same method. Given that the bioactive compounds’ loading method involves grinding the 5-FU/phenolic compound solutions with magnetite powders until the evaporation of the solvent, the drug loading efficiency is maximum [56]. 

### 2.3. Characterization of the Magnetic-Based Systems

The MNPs loaded with bioactive compounds were characterized using specific analysis that ensured the establishment of specific properties. 

Transmission Electron Microscopy (TEM) and High-Resolution TEM (HR-TEM) images and SAED pattern were acquired on a high-resolution 80–200 TITAN THEMIS transmission microscope (purchased from FEI, Hillsboro, OR, USA). The microscope was equipped with a column EDXS detector and an image corrector, and it was operated in transmission mode at a voltage of 200 kV. In total, 10 μL deionized (DI) water were placed into a 400-mesh lacey carbon-coated copper grid. The size of the NPs was measured with the help of ImageJ software (version 1.8.0, National Institutes of Health, Madison, WI, USA) [4].

X-ray diffraction (XRD) was performed using a Shimadzu XRD-6000 diffractometer (Shimadzu Corporation, Kyoto, Japan) equipped with a Cu Kα radiation source. Scans were obtained between 20° and 80° 2θ with an acquisition step of 0.02. The crystallite sizes were calculated using the Scherrer equation.

The magnetic properties of the developed MNPs were evaluated with the vibrating sample magnetometer at 25 ± 2 °C using the 7400 Series VSM equipment manufactured by LakeShore (Lakeshore, CA, USA) [55].

The determination of the size, morphology, and elemental composition of MNPs were assessed using a Quanta Inspect F50 (FEI Company, Eindhoven, The Netherlands) scanning electron microscope equipped with a field emission gun electron (FEG) with 1.2 nm resolution and an Energy Dispersive X-ray spectrometer with a MnK resolution of 133 eV [55].

The Fourier Transform Infrared Spectroscopy (FT-IR) spectra were recorded at room temperature in ATR mode using a Nicolet iS50 spectrometer (Thermo Fischer Scientific, Waltham, MA, USA). The spectrum of each sample is an average of 64 scans, in the 400–4000 cm^–1^ range, at 4 cm^–1^ resolution [57]. 

Dynamic Light Scattering (DLS) measurements were performed after dispersing 5 mg of the developed MNPs in 15 mL of phosphate-buffer saline (PBS) 1× solution with a pH of 7.4 and dispersed using a Sonorex Digitec DT 514 ultrasonic bath (Bandelin, Berlin, Germany) for 10 min at 25 °C. A small amount of the dispersion was further introduced into the measurement cell and placed inside the DelsaMax Pro equipment (Backman Coulter, Brea, CA, USA). Three measurements were performed for each sample [4].

The thermogravimetric analysis (TG-DSC) was performed with a STA 449 F3 Jupiter equipment from Netzsch (Selb, Germany). About 10 mg of dry powder was placed in an alumina crucible and heated up to 900 °C with a 10 °C min^−1^ rate under a flow of 50 mL min^−1^ of dried air [58]. 

### 2.4. Antitumoral Agent Release Behavior

The 5-FU release profile was performed using the dialysis bag method [4,59]. Quantification of 5-FU was performed by plotting a calibration curve in the 0.1–10 μg/mL range. After that, 20 mg MNPs@5-FU 2%, respectively, 40 mg MNPs@5-FU 1%, were placed inside a dialysis bag, followed by immersion in 40 mL PBS 1× (pH = 7.4). The samples were slowly shaken in an orbital motion at 25 rpm for 48 h at 37 °C in a thermoshaker (Gerhardt GmbH & Co., Königswinter, Germany). At different time points, 500 μL of supernatant were collected and replaced with fresh PBS. The measurements were performed at 266 nm wavelength using a Thermo Evolution 600 double-beam UV-Vis Spectrophotometer (Thermo Fisher Scientific, Waltham, MA, USA) and a 1 cm optical path quartz cuvette. The 5-FU concentration was calculated using the calibration curve, and the cumulative release percentage (%) was determined with Equations (1) and (2), when *P_t−_*_1_ is the percentage release at the *t* − 1 time point, and *P_t_* is the percentage release at the t time point.

(1) $$Drug\;release\left(\%\right)=\frac{amount\;of\;drug\;in\;release\;medium}{amount\;of\;drug\;loaded\;on\;MNPs}\times 100$$

(2) $$Cumulative\;drug\;release\;\left(\%\right)=\frac{volume\;of\;sample\;withdrawn}{volume\;of\;release\;medium}\times P_{t-1}+P_{t}\;$$

Table 1 presents the mathematical models that were performed to study the release kinetics of the 5-FU-loaded MNPs [60,61]. 

The drug release data results were performed using Microsoft Excel 365 (Microsoft Corporation, Redmond, OR, USA) and GraphPad Prism 10.2 (San Diego, CA, USA). 

### 2.5. Phenolic Compounds Release Profiles

The PCs’ release profile was performed using the dialysis bag method previously described. In total, 40 mg MNP were placed inside a dialysis bag followed by immersion in 40 mL PBS 1× (pH = 7.4), and the samples were slowly orbitally shaken at 25 rpm for 120 h at 37 °C. At different times, 250 μL of supernatant were collected and replaced with fresh PBS. The total phenol content (TPC) was achieved utilizing the Folin-Ciocalteu method [43,62]. A total of 125 μL of sample or standard (gallic acid), 1.25 mL of Folin-Ciocalteu reagent (diluted 10 times in DI), and 1 mL of 1 M of sodium carbonate were homogenized, and the absorbance was measured after 15 min (min) at 746 nm. A calibration curve was plotted with standard solutions of gallic acid with concentrations varying between 0.125 and 6 mg/L (R^2^ = 0.9979). TPC was expressed as a cumulative percentage using Equations (1) and (2). Furthermore, the previously described mathematical models (Table 1) were performed to study the release kinetics of PCs. 

### 2.6. Antioxidant Activity

#### 2.6.1. Determination of Free-Radical Scavenging Capacity (DPPH)

Free radical scavenging activity of loaded MNPs was performed by using a spectrophotometric analysis [13,63]. DPPH (7 mg) was used as a free radical, which was dissolved in ethanol (30 mL) and vortexed until solubilization. Several concentrations of MNPs (1.25–10 mg/mL) were diluted in DI water, and in a 96-well plate were added 20 μL of MNPs suspension and 180 μL DPPH solution. The plate was incubated for 30 min in the dark, and spectrophotometric measurements were performed at 518 nm. Also, the DPPH assay was performed for extracts (20 μL BPEs/BBEs were mixed with 180 μL DPPH solution and performed the same method previously described). The experiment was performed in triplicate, and the percent of DPPH inhibition was calculated using Equation (3).

(3) $$DPPH\;Inhibition\left(\%\right)=\frac{A-B}{A}\times 100\;$$

A is represented by the absorbance of the oxidized solution in the absence of antioxidant agents (control—DPPH), and B is the absorbance of the oxidized solution in the presence of antioxidant agents. The concentration required to inhibit 50% of the DPPH radical (IC_50_) was assessed using linear regression acquired using the plotting concentration vs DPPH inhibition (%). 

#### 2.6.2. Ferric Reducing Antioxidant Power (FRAP) Method

The reaction mixture included 50 μL of sample/standard and 950 μL of FRAP regent [64]. A UV-VIS spectrophotometer (Mulsiskan FC instrument, Thermo Scientific, Waltham, MA, USA) was used to detect the absorbance at λ = 593 nm after 25 min of dark incubation at 37 °C and 5 min of spinning at 7000 rpm. The calibration curve was plotted using 1 mM Trolox stock solution at concentrations ranging from 30 to 250 μM (R^2^ = 0.9987).

#### 2.6.3. Cupric Reducing Antioxidant Capacity (CUPRAC) Assay

The reaction mixture included 240 μL of sample/standard solutions of different concentrations, 200 μL CuCl_2_ (10 mM), 200 μL neocuproin (7.5 mM), and 200 μL ammonium acetate buffer 1 M, pH = 7.00 [65]. After 30 min, the absorbance was measured at 450 nm. The concentrations used for the Trolox calibration curve ranged from 0.125 to 1.5 mM (R^2^ = 0.9943).

### 2.7. Biological Activity of Magnetic-Based Systems

The biological properties of developed DDSs were assessed using standard Gram-positive and Gram-negative bacteria (*Enterococcus faecalis* ATCC 29212, *Staphylococcus aureus* ATCC 25923, *Escherichia coli* ATCC 25922, and *Pseudomonas aeruginosa* ATCC 27853), and a probiotic bacteria (*Lactobacillus rhamnosus* MF9) isolated from newborn feces. A cell viability assay was performed using a cell line specific for CRC (HT-29). To ensure the sterility of the experiments, MNP-based samples were sterilized for 30 min of UV radiation. 

#### 2.7.1. Qualitative Evaluation of Antibacterial Activity

The qualitative antibacterial activity was performed using an adapted spot diffusion method, according to the Clinical Laboratory Standards Institute [66,67]. The bacterial suspensions corresponding to 1.5 × 10^8^ CFU/mL were prepared from 24 h cultures on NB with agar media. A stock of MNPs (100 mg/mL) suspension prepared with sterile physiological buffer saline (PBS) was used. Petri plates with NB media were seeded with inoculums, and 20 µL of each sample was spotted. After diffusion, the dishes were incubated at 37 °C for 24 h. 

#### 2.7.2. Quantitative Evaluation of Antibacterial Activity

The minimum inhibitory concentration (MIC) assay used an adapted binary serial microdilution standard assessment in NB medium [66]. In a 96-well plate, for each MNP sample, serial two-fold micro-dilutions were performed in 150 µL of broth medium seeded with the standard inoculum. The plates were incubated at 37 °C for 24 h. Visual and spectrophotometric analyses determined the MIC values by measuring the absorbance at 620 nm using the BIOTEK SYNERGY-HTX ELISA multi-mode reader (Winooski VT, USA) [66].

#### 2.7.3. Semiquantitative Assessment of Microbial Adherence to the Inert Substratum

The biofilm development on the inert substratum was determined using the same serial two-fold microdilution method [66]. After 24 h of incubation and MIC measurements, the media from plates was removed, the walls were washed three times with PBS, and the bacterial cells adhered to the walls were fixed with methanol (5 min) and tinted with 1% CV (15 min). The dyed biofilm was resuspended with 33% AcA, and the absorbance was measured at 490 nm [66]. 

#### 2.7.4. Evaluation of the Influence of the Magnetic-Based Systems on the Growth of a Microbial Strain with Probiotic Potential

The MNPs influence on the growth of *L. rhamnosus* MF9 was evaluated by following its growth and multiplication for 24 h using bacterial suspension, which corresponded to 1.5 × 10^8^ CFU/mL and was prepared in PBS from fresh culture (24 h) [42]. The influence of MNPs against probiotic bacteria was evaluated in liquid media. In sterilized Eppendorf Safe-Lock tubes were performed binary-serial dilutions from 1000 to 125 μg/mL MNPs, followed by inoculation of *L. rhamnosus* MF9. The volumetric ratio between the volume of inoculated microbial suspension and of the broth media = 1:10. The tubes were incubated at 37 °C in anaerobic conditions, and the growth was evaluated after 24 h of incubation. Serial dilutions from each sample were inoculated on a MRS agar plate in triplicate, and viable cell counts were assessed after 24 h incubation in anaerobic conditions at 37 °C, and are expressed as CFU/mL. Also, in 96-well plates were transferred 100 μL from tubes, and spectrophotometric measurements were performed at λ = 600 nm. The experiments were performed in triplicate. The results were expressed in percentages and calculated using Equation (4).

(4) $$L.\;rhamnosus\;MF9(\%)=\frac{A600\;sample}{A600\;control}\times 100\;$$

#### 2.7.5. The Antiproliferative Assay

The HT-29 cells were cultured in Dulbecco’s Modified Eagle’s Medium (Gibco, Boston, MA, USA) supplemented with 10% fetal bovine serum (Gibco, Boston, MA, USA), 100 U/mL penicillin, and 100 μg/mL streptomycin, in a humidified atmosphere (with 5% CO_2_) at 37 °C. The culture medium was changed every 2 days until cells reached confluence, and then the cells were detached with 0.25% trypsin–0.53 mM EDTA (Sigma-Aldrich, Darmstadt, Germany). For the experiments, cells were seeded at 5 × 10^4^ cells/cm^2^ and left overnight to adhere. Cellular viability was measured using the 3-(4,5-dimethylthiazol-2-yl)-2,5-diphenyltetrazolium bromide (MTT; Sigma-Aldrich, Darmstadt, Germany) assay. At the end of the incubation periods, the culture medium was removed, and the cells were incubated with 1 mg/mL MTT for 2 h at 37 °C. The purple formazan crystals formed in the viable cells were dissolved with 2-propanol (Sigma-Aldrich, Darmstadt, Germany), and the absorbance was measured at 595 nm using a plate multi-reader (FlexStation 3, San Jose, CA, USA) [68]. The cell viability of the samples was calculated using Equation (5).

(5) $$Cell\;viability\left(\%\right)=\frac{A595\;sample}{A595\;control}\times 100$$

### 2.8. Statistical Analysis

The data results were statistically analyzed using GraphPad Prism 10.2 from GraphPad Software (San Diego, CA, USA). The data results are expressed as ±SD (standard deviation) and analyzed using a one-way analysis of variance (one-way ANOVA) and Tukey’s/Holm-Šídák’s multiple comparisons test. The differences between samples were considered statistically significant when the *p*-value was < 0.05.

## 3. Results and Discussion

### 3.1. Magnetic-Based Systems Characterization

In the present study, the synthesized citrate-coated MNPs were first loaded with the BPEs and BBEs, which presented the highest concentration of PCs and antioxidant and antimicrobial properties determined previously [42,43]. Additionally, an antitumoral agent (5-FU) was loaded on the surface of Fe_3_O_4_@BPE and Fe_3_O_4_@BBE NPs to alleviate the adverse effects on the gut microbiota. In this approach, the developed nanocarriers were characterized using TEM, SEM, EDX, XRD, VSM, FT-IR, DLS, and thermal analyses. 

#### 3.1.1. Transmission Electron Microscopy

**Figure 2:**
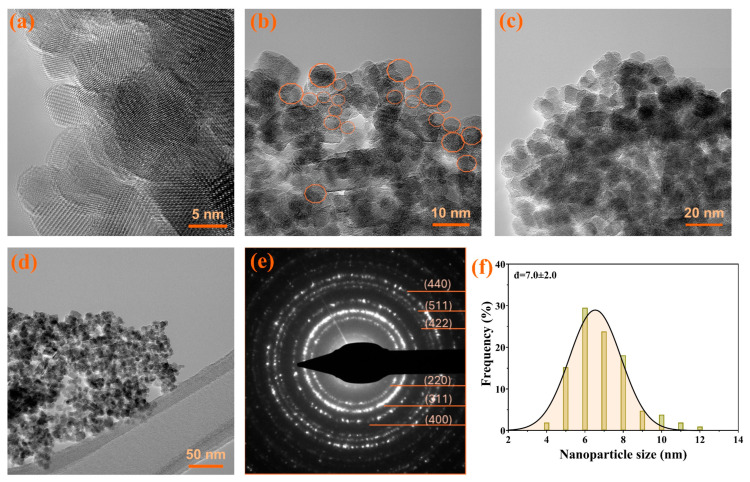
The TEM images, SAED pattern, and size distribution for citrate-coated Fe_3_O_4_ NPs. (**a**–**d**) HR-TEM, (**e**) SAED patterns, (**f**) size distributions. The quasi-spherical shapes were marked with orange circles in figure (**b**).

The morphology of the citrate-coated MNPs was evaluated using the TEM, HR-TEM, and SAED assessments (Figure 2). As can be observed, the NPs have a quasi-spherical shape and possess an increased tendency to agglomerate, probably due to the magnetic dipole moment interaction between particles. Moreover, these images were used to determine the size distributions of the MNPs. The distributions are considerably narrow, suggesting that the size of the MNPs is between 5 and 10 nm (average size particle of 7.0 ± 2 nm), which could further demonstrate their usability in the desired/targeted applications. Furthermore, the SAED results confirm that the patterns are linked with the Miller indices characteristic for Fe_3_O_4_ [69,70]. 

#### 3.1.2. Scanning Electron Microscopy and Energy Dispersive X-ray Assay

**Figure 3:**
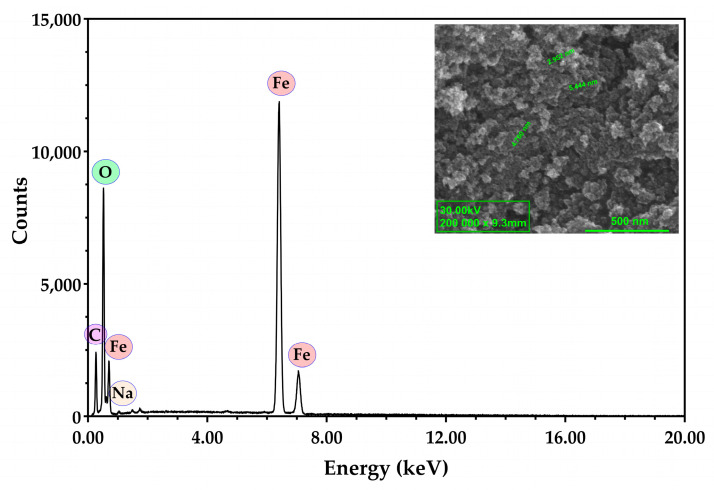
Representative SEM image and EDX analysis for citrate-coated Fe_3_O_4_ NPs.

The citrate-coated MNPs present a quasi-spherical shape with diameters less than 10 nm, and possess a tendency to agglomerate, probably due to the magnetic dipole moment interaction between particles (Figure 3). EDX confirms the stabilization of Fe_3_O_4_ NPs with citrate by the presence of the additional element on the MNPs’ surface (C). Also, it is important to mention that the citrate shell leads to an overall negative charge (see also DLS data in Section 3.1.7) of the MNPs, while Na is present only in a small amount.

#### 3.1.3. VSM Analysis

**Figure 4:**
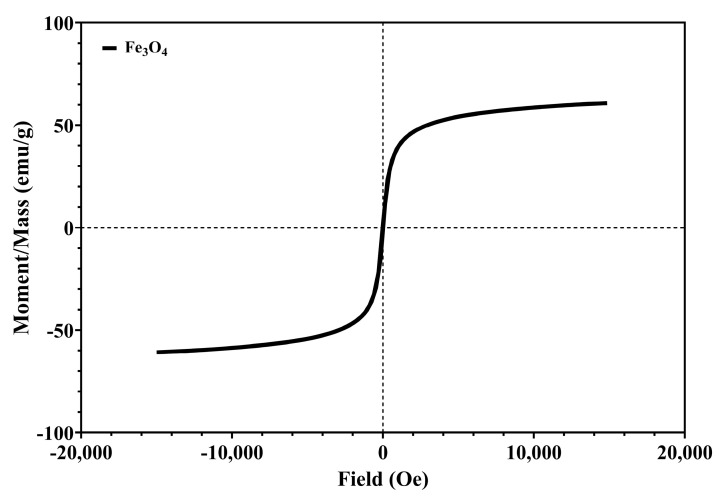
The magnetization/mass ratio for citrate-coated Fe_3_O_4_ NPs.

The analysis of the degree of the magnetization indicates the characteristic magnetization curve that corresponds to citrate-coated MNPs (Figure 4). 

Figure 4 presents a symmetric hysteresis curve, and indicates the superparamagnetic behavior of citrate-coated MNPs. The coercivity (Hci) of the citrate-coated MNPs is 13.639 Oe and saturation magnetization (Ms) is 60.506 emu/g, which are correlated with other research with similar synthesis [71,72]. The Ms of bulk magnetite is around 90 emu/g, and a lower value is attributed to the small particle size effect and the sodium citrate layer [72]. 

#### 3.1.4. X-ray Diffraction

**Figure 5:**
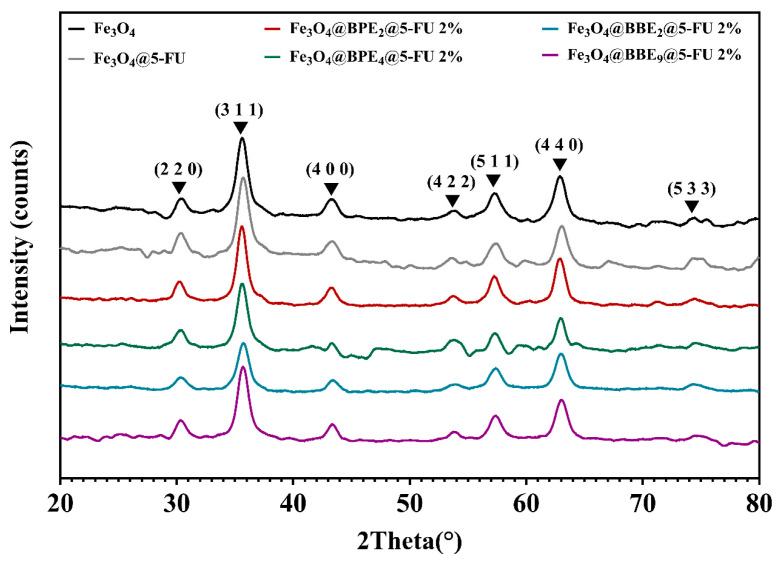
Diffractograms for magnetite and loaded MNPs.

The structure of the obtained nanocarriers was determined using X-ray diffraction, which confirmed the cubic structure of MNPs for all samples (Figure 5). Consequently, adding bioactive compounds from extracts and 5-FU does not conduct the formation of secondary iron oxides. 

Figure 5 displays the characteristics peaks of tetrahedral and polyhedral magnetite crystals as follows: 30.2°, 35.6°, 43.2°, 53.6°, 57.2°, 63°, and 74.3° [55,73]. Table 2 displays the crystallite size for the obtained MNPs, which are expressed as mean value ± standard deviation (SD).

The crystallite sizes of developed MNPs range from 6.19 ± 0.89 to 8.31 ± 2.49 nm. Based on the presented results, it can be concluded that the ethanol used in the extract preparation and for 5-FU solubilization did not influence the magnetic structure of the MNPs.

#### 3.1.5. FT-IR Spectroscopy

A FT-IR analysis was performed to establish the bonds and functional groups present within the obtained MNPs.

**Figure 6:**
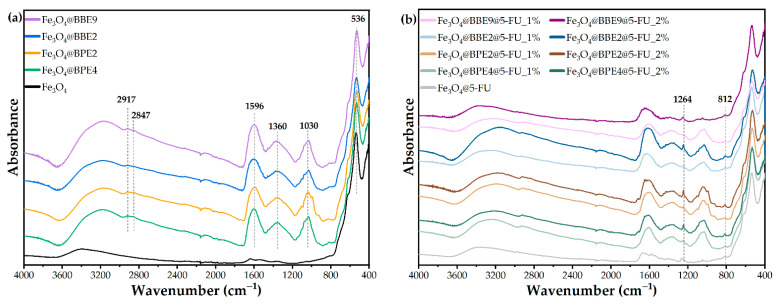
FT-IR spectra for magnetite and loaded MNPs. (**a**) MNPs with extracts and (**b**) MNPs with extracts, and antitumoral drug.

The FT-IR spectrum of the bare Fe_3_O_4_ sample (Figure 6a) exhibits distinctive peaks consistent with the molecular structure of magnetite (Fe_3_O_4_). The prominent absorption band at approximately 536 cm^–1^ indicates the Fe–O bond-stretching vibrations, a defining characteristic of magnetite. Moreover, the broad peak centered around 3400 cm^–1^ is attributed to O–H stretching vibrations, likely from absorbed water or hydroxyl groups on the sample’s surface. Similar FT-IR patterns of bare Fe_3_O_4_ have been extensively reported in the literature, including prior studies conducted within our research group [4,55]. Fe_3_O_4_ NPs coated with BPEs and BBEs similarly displayed the characteristic peaks of Fe–O stretching vibrations at the same wavelengths. Nevertheless, the broad peaks attributed to O–H vibrations shifted to shorter wavenumbers, around 3180 cm^–1^, and could be ascribed to O–H stretching vibrations from both the extracts and the residual water molecules on the surface of the NPs. By comparison, the respective peaks also changed their shape, becoming more pronounced and broader, possibly because of N-H stretching vibrations present in proteins [74]. Apart from the absorption bands corresponding to magnetite, the spectra are comparable to those obtained in our previous study [43], in which we evaluated bee bread extracts from a chemical and microbiological perspective. Precisely, additional peaks in the coated NPs arise from specific functional groups present in the bee pollen and bee bread extracts, such as at 2917 cm^–1^ and 2847 cm^–1^, corresponding to the symmetric and asymmetric stretching of C–H groups present in carbohydrates and lipids or the large peak at 1030 cm^–1^ corresponding to C–O and C–OH vibrations in carbohydrates and PCs [75]. 

Concerning the samples loaded with 5-FU, Figure 6b shows the preservation of the above-mentioned signals, common to magnetite, BP, and BB extracts. Absorption bands at 1264 cm^–1^ and 812 cm^–1^, corresponding to C–H stretching (in-plane) and C–F stretching in 5-FU [76], were observed in the magnetite sample loaded with the active substance (Fe_3_O_4_@5-FU). The signals were further distinguished for the drug-loaded samples coated with the extracts, and it was noticeable that the intensity of these bands varied with concentrations, visibly more intense in the case of samples loaded at higher concentrations of 5-FU (2% versus 1%).

#### 3.1.6. Thermal Analysis 

**Figure 7:**
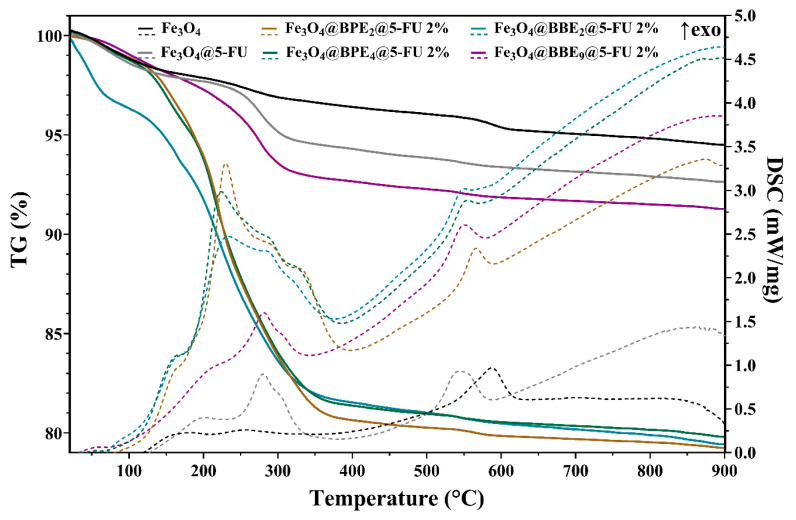
TG-DSC curves for obtained MNPs.

The amount of loaded bioactive compounds was assessed using the TG-DSC assay (Table 3). In all MNP samples, under 200 °C, adsorbed water and volatile molecules are lost, with the associated effect on the DSC curve being endothermic. The organic compounds from bee product extracts loaded on the MNPs start to degrade, mainly by oxidation, after 100–150 °C, and Fe(II) is oxidized to Fe(III) when Fe_3_O_4_ is transformed to maghemite (γ-Fe_2_O_3_) [58,77]. The maximum exothermic effect on the DSC curve is around 223.1–233.1 °C, except Fe_3_O_4_@BBE_9_@5-FU 2%, which occurs at 279.6 °C. The asymmetric shape of the effect indicates the existence of multiple oxidative processes up to 400 °C. The phase transformation of maghemite to hematite (α-Fe_2_O_3_) occurs around 550 °C, as indicated by the corresponding exothermic effect on the DSC curve. The residual mass, reddish-brown, consists of α-Fe_2_O_3_ [78,79]. The primary data from thermal analysis are presented in Figure 7 and Table 3.

#### 3.1.7. DLS Assay

**Figure 8:**
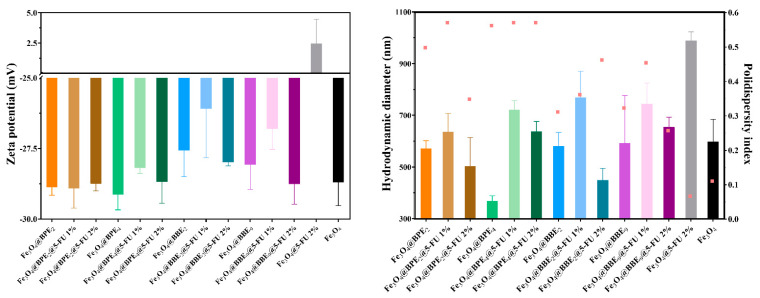
The zeta potential, hydrodynamic diameter (illustrated as columns), and the PDI (illustrated as pink dots) values for obtained MNPs.

The stability of the nanocarriers was evaluated through zeta potential, hydrodynamic diameter, and polydispersity index (PDI) measurements (Figure 8). The hydrodynamic diameter increases when it is loaded with 1% 5-FU, but decreases when it is added with a 2% anticancer agent. It can be assumed that the 5-FU is hydrophilic, and its addition at small concentrations increases the MNPs’ hydrophilicity. In contrast, 5-FU overlaps the functional groups from BPEs/BBEs at higher concentrations and decreases the interaction with the solvent (PBS). For this reason, the Fe_3_O_4_@5-FU 2% sample presented a higher hydrodynamic diameter value. According to the zeta potential values for Fe_3_O_4_@5-FU 2%, Fe_3_O_4_@BBE_2_@5-FU 1%, and Fe_3_O_4_@BBE_9_@5-FU 1%, and to the higher hydrodynamic diameter, values may be due to the agglomeration of NPs. Considering the PDI values that are lower than 0.6, they indicate a thin/narrow particle distribution. The smaller PDI values indicate the long-term stability and uniformity [80,81,82]. 

### 3.2. Bioactive Agents’ Release Behavior

**Figure 9:**
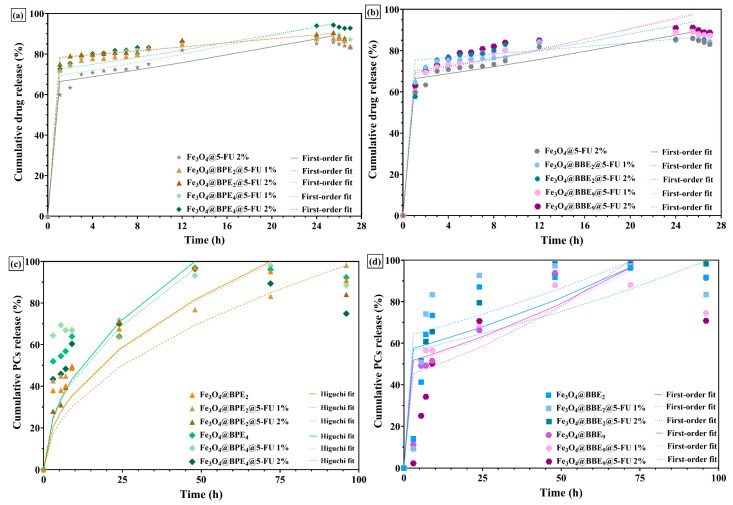
Antitumoral drugs’ and PCs’ release profiles of nanocarriers loaded with BPEs (**a**,**c**) and BBEs (**b**,**d**).

5-FU, a pyrimidine-antineoplastic agent, inhibits the enzyme thymidylate synthase, deoxyribonucleic acid (DNA), and ribonucleic acid (RNA) synthesis, which may lead rapidly to cell death. According to the Food and Drug Administration (FDA) and the European Medicines Agency (EMEA), 5-FU should be administered intravenously [83,84]. Therefore, the drug and PCs release profiles (Figure 9) were assessed in PBS (pH = 7.4) at 37 °C. 

The 5-FU drug release profiles of nanocarriers loaded with BPEs and BBEs showed that the antitumoral drug releases rapidly in the first hour, but at a lower rate for samples without extracts (Figure 9a,b). Also, it can be observed that in two hours, the 5-FU has a maximum/fast release rate (~75% released), followed by a plateau. The nanocarriers with 5-FU and extracts showed a higher percentage of drug released after 24–25.5 h, and a slower release of drug molecules/degradation can be observed immediately. Likewise, Fe_3_O_4_@5-FU presented a similar profile/pattern, but with a smaller percentage released. It could be assumed that biomolecules from extracts facilitated the antitumoral drug to release in higher amounts in the first hours, which can be helpful for the death of tumoral cells [85,86]. Conversely, the PCs’ release profiles (Figure 9c,d) illustrate similar patterns with prolonged release rates. In both cases, the PCs are gradually released from MNPs, reaching a plateau after 48 h, maintained up to 96 h, followed by a decrease that suggests the PCs’ degradation. The higher release time of PCs compared to 5-FU is attributed to the flavonoids in extracts. These results are in agreement with another study [87], which reported similar prolonged release behavior for MNPs with quercetin. Moreover, since the surface of MNPs was firstly loaded with extracts and followed by 5-FU, the loading of the drug did not slow/affect the PCs’ release. 

In a recent study [60], magnetic nanocomposites were used as carriers for 5-FU and curcumin, and the release percentages for the anticancer drug in PBS ranged from 55 to 59% and 31 to 40% for curcumin in the first 24 h (at 37 °C). The shape of the release curves is similar to those obtained in our study, but the availability of 5-FU in the first 6 h from MNPs is higher. Also, the PCs’ release rates are comparable to curcumin profiles, and from our knowledge, there are no literature data to confirm the release profiles of PCs, which are derived from BPEs/BBEs and are loaded on MNPs or another matrix. 

Table 4 represents the kinetic parameters of four mathematical models applied to obtain the release data’s best-fit mechanism/release profile. 

The experimental data were subjected to four mathematical models (zero-order, first-order, Higuchi, and the Hixson–Crowell model). The zero-order kinetics describe that the constant release of 5-FU is independent of its concentration, and is considered the ideal model. Still, in our case, the regression coefficient (R^2^) values are the smallest. According to Table 4 and the kinetic parameters for each model, the best-fit model for drug release is the first-order model, which is correlated with the hydrophilic molecules. However, the Fe_3_O_4_@BBE_2_@5-FU 1% and Fe_3_O_4_@BBE_2_@5-FU 2%, the R^2^ values are less than 0.900, a fact that is unacceptable, and these samples are not suitable for pharmaceutical formulations and in vivo applications. 

In the case of the PCs’ release kinetics, the MNPs loaded with BPEs are linked to the Higuchi model, which is correlated to low solubility in the solid matrix, respectively, to a layer on the surface of the MNPs that prevents the release of the PCs rapidly over time [88]. Otherwise, the highest R^2^ values of MNPs loaded with BBEs are associated with the first-order release, but for some samples, such as Fe_3_O_4_@BBE_2,_ Fe_3_O_4_@BBE_2_@5-FU 1%, and Fe_3_O_4_@BBE_9_@5-FU 1%, the regression coefficient values for all applied mathematical models are significantly lower. 

### 3.3. Antioxidant Activity

**Figure 10:**
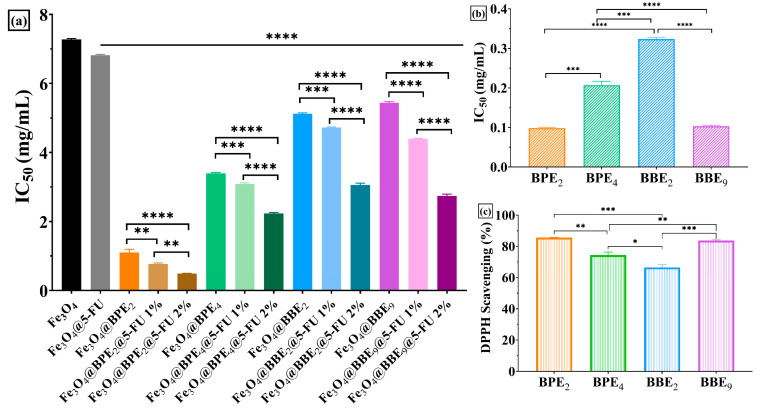
Antioxidant activity of developed MNPs using the DPPH assay. (**a**) IC_50_ values for nanocarriers, (**b**) IC_50_ values for extracts, and (**c**) DPPH% for extracts. The data results are considered statistically significant (* *p* < 0.013, ** *p* < 0.07, *** *p* < 0.008, **** *p* < 0.0001).

The DPPH assay is a spectrophotometric assessment based on the antioxidants’ capability to scavenge the chromogen radical. The formation of reduced DPPH radicals, due to radical reduction by hydrogen atom transfer from antioxidants, induces changes in the color of the solution from violet to light yellow [89]. Different concentrations of MNPs were assessed to establish the antioxidant concentration that scavenges 50% of the initial DPPH radical, which is known as IC_50_ (Figure 10). The lower IC50 values correspond to higher antioxidant activity [90]. 

The developed MNPs presented the capacity to scavenge the DPPH radical, as expected, considering the previous studies on BPE and BBE antioxidant activity (TEAC method) [42,43]. Figure 10 highlights the lower IC_50_ values for MNPs loaded with BPEs and BBEs than Fe_3_O_4_@5-FU 2% or Fe_3_O_4_ samples. Moreover, it can be observed that a significant difference between samples with BPEs and BBEs and smaller IC_50_ values for MNPs loaded with BPE_2_ indicate that the obtained nanocarriers are more potent antioxidants that could induce lower toxicity for patients [13]. The data results obtained using the DPPH assay confirmed the antioxidant activity of the extracts (Figure 10b,c), except MNPs loaded with BBE_9_, which can scavenge the radical more than Fe_3_O_4_@5-FU 2%. The lower antioxidant activity for this sample could be explained by smaller amounts of bioactive compounds loaded on the MNPs’ surface, according to thermal analysis results (Table 3). Additionally, Figure 10a displayed a decrease in IC_50_ values simultaneously with the concentration of 5-FU loaded on the MNPs, which implies a synergic effect between the antitumoral drug and PCs from extracts. Similar results are reported for Fe_3_O_4_ loaded with PCs (gallic acid, quercetin, and plant extracts) [13,63,91]. 

Furthermore, the antioxidant activity of synthesized MNPs was also evaluated using the FRAP and CUPRAC methods. Both the FRAP and CUPRAC assays are methods based on electron transfer (ET). CUPRAC offers different advantages over other ET-based tests, namely working conditions at physiological pH, the ability to quantify both hydrophilic and lipophilic antioxidants, selectivity oxidation of antioxidant compounds without interfering with sugars and citric acid, and it can quantify the SH-class antioxidants [92]. As a principle, the compound Cu(II) bis-neocuproine (Cu(II)Nc) reacts with the donor groups of electrons in the antioxidant molecule, and it is reduced to the complex Cu(I) Bis-neocuproin (Cu(I)NC) (the reduction potential of Cu(II)-NC/Cu(I)-NC is 0.6V) [93].

**Figure 11:**
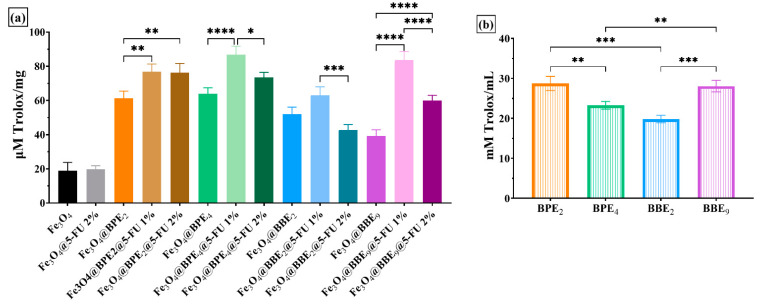
Antioxidant activity of developed MNPs using the CUPRAC assay for nanocarriers (**a**) and extracts (**b**). The data results are considered statistically significant (* *p* < 0.05, ** *p* < 0.01, *** *p* < 0.001, **** *p* < 0.0001).

The antioxidant activity evaluated using the CUPRAC method highlighted that all MNPs with 5-FU 1% showed a better antioxidative activity compared to their counterparts with 5-FU 2% and without the antitumoral agent, respectively, except for the Fe_3_O_4_@BPE_2_@5-FU 1% /Fe_3_O_4_@BPE_2_@5-FU 2% where the differences are insignificant (*p* > 0.05). It can be concluded that as the concentration of 5-FU increases, the antioxidant activity decreases. Among the groups tested, as shown in Figure 11a, the MNPs with BBE_2_ showed the weakest activity, following the trend in the extract’s activity (Figure 11b). All of the extracts’ antioxidant activities were significantly higher than Fe_3_O_4_@ and Fe_3_O_4_@5-FU 2% (*p* < 0.0001) (Figure 11a).

FRAP evaluates hydrophilic antioxidants resistant to acid pH (pH = 3.6), which are not protein based (proteins precipitate at pH < pI) and do not have thiol groups [94]. In the FRAP method, the ET agent is the Fe^3+^. The reduction potential of the redox couple Fe^3+^/Fe^2+^ is 0.771V. At the time of the reaction, the Fe^2+^ ions form with TPTZ, a complex that can react with various oxidable groups [95]. 

**Figure 12:**
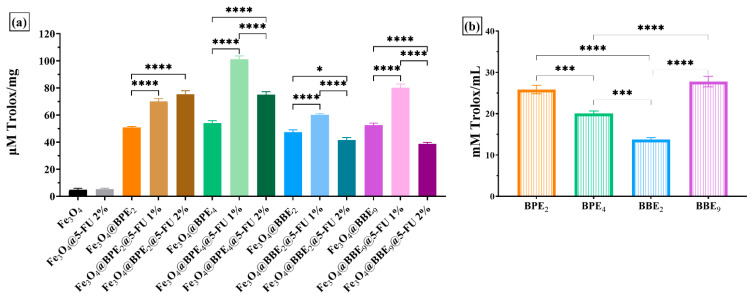
Antioxidant activity of developed MNPs using the FRAP assay for nanocarriers (**a**) and extracts (**b**). The data results are considered statistically significant (* *p* < 0.05, *** *p* < 0.001, **** *p* < 0.0001).

The antioxidant activity evaluated using the FRAP method highlighted the same trend as the CUPRAC method, namely that all MNPs with 5-FU 1% showed a better antioxidant activity compared to their counterparts with 2-FU 2% and without the antitumoral agent, respectively, except for the Fe_3_O_4_@BPE_2_@ 5-FU 1%/Fe_3_O_4_@BPE_2_@5-FU 2%, where Fe_3_O_4_@BPE_2_@5-FU 2% showed a better activity but were statistically insignificant (*p* > 0.05). Among the groups tested, it can be seen from Figure 12a that the group with BBE_2_ presents lower activity in accordance with the trend in the extracts’ activity (Figure 12b). The best activity was for the BPE_4_ sample, although from Figure 12b, the activity was weaker compared to BPE_2_ (*p* < 0.001) and BBE_9_ (*p* < 0.0001).

Various methods were applied [13,63] to measure the total antioxidant capacity of magnetite nanocarriers, which mainly differ in their chemical reaction for producing different radicals and/or targeting molecules, as well as in measuring endpoints [96].

The combined use of DPPH, FRAP, and CUPRAC methods ensures a comprehensive analysis of the antioxidant capacity, providing detailed and relevant information for various potential applications. Thus, if the first method shows the ability to scavenge DPPH free radicals, CUPRAC details the chelating capacity of copper ions, while FRAP provides data on the metal-reducing capacity of MNPs. Applying these in vitro methods simultaneously closely reflects the in vivo action of MNPs’ antioxidant capacity [97].

The antioxidant activity of the MNPs with BPE/BBE extracts is linked to the PC profiles previously established by UHPLC-DAD-ESI/MS [42,43]. Thus, BPE_2_ contained high amounts of rutin, 4-hydroxybenzoic acid, and ferulic acid, while BPE_4_ likewise contained a high content of rutin, chlorogenic acid, quercetin, and kaempferol. The antioxidant activity of BBE_2_ and BBE_9_ could mainly be attributed to quercetin, kaempferol, isorhamnetin, rutin, resveratrol, and caffeic acid. All PCs present strong antioxidant activity [98,99]. 

### 3.4. Antibacterial Activity

#### 3.4.1. Qualitative Assessment

**Figure 13:**
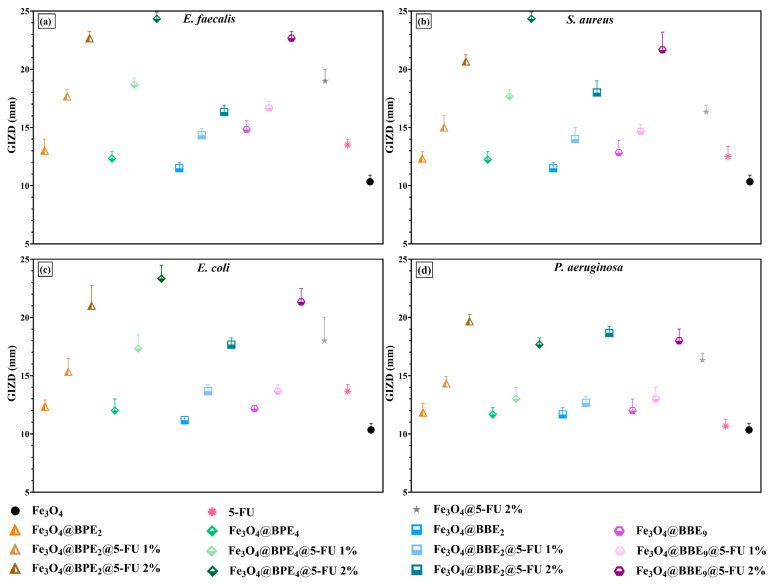
The representation of GIZDs: (**a**) *E. faecalis*, (**b**) *S. aureus*, (**c**) *E. coli*, and (**d**) *P. aeruginosa*. The differences between MNPs were statistically analyzed using the one-way ANOVA followed by Tukey’s multiple comparisons tests, and the data were considered statistically significant.

Firstly, the antibacterial properties of based MNPs were qualitatively evaluated by measuring the growth inhibition zone diameters (GIZDs) that developed near the spot, expressing them as mean values ±SD (Figure 13).

The antimicrobial profiles of developed MNPs highlight a significant inhibitory effect on the growth of tested Gram-positive and Gram-negative bacteria **(**Figure 13). The MNPs displayed the highest sensitivity on *E. faecalis*, *S. aureus,* and *E. coli*, followed by *P. aeruginosa*. Overall, the inhibitory effect increased with the quantity of bioactive compounds loaded on the surface of MNPs, and the highest inhibition was obtained for samples with 2% 5-FU. Moreover, the MNPs loaded with BPEs/BBEs and antitumoral drugs determined a higher sensitivity than MNPs loaded only with 5-FU (Fe_3_O_4_@5-FU 2%), which could be due to a synergistic effect between PCs from extracts and 5-FU. As well, recent research [60] reported the enhancement of the antibacterial activity of MNPs with 5-FU by adding curcumin. 

The highest antibacterial activity on Gram-positive bacteria was for MNPs loaded with BPE_4_, followed by BBE_9_ and BPE_2_. Also, the MNPs with BBE_2_ presented significant inhibitory effects/properties. In the case of *E. coli* and *P. aeruginosa*, the MNPs showed similar antimicrobial profiles, but MNPs loaded with BPE_2_ determined the highest sensitivity. The antibacterial activity of BPE-loaded MNPs is confirmed by the inhibitory effect of the extracts on the tested strains. *E. coli* and *P. aeruginosa* were the most sensitive strains on the influence of BPE_2_, and likewise, *S. aureus* on BPE_4_ [42]. Also, BBE_2_ and BBE_9_ presented higher GIZD values on Gram-positive and Gram-negative bacteria [43]. 

Gram-negative bacteria are more resistant to ATBs than Gram-positive bacteria due to their outer membrane [100], but iron oxide NPs can penetrate the outer and inner membrane of the cell wall due to the reactive oxidative species’ (ROS) generation/excessive production, capacity to break mercapto, amino, and carboxyl groups of proteins, and inhibition of DNA replication [101]. In a research [102], various concentrations of Fe_3_O_4_ NPs (25–100 μg/mL) determined a significant inhibitory effect on *E. coli* and *Proteus vulgaris*, with GIZD ranging from 11 to 21 mm. The MNPs have 33–40 nm size, and also inhibited *S. aureus* and *Xanthomonas* (8–11 mm). 

#### 3.4.2. Minimum Inhibitory Concentration Assay

**Figure 14:**
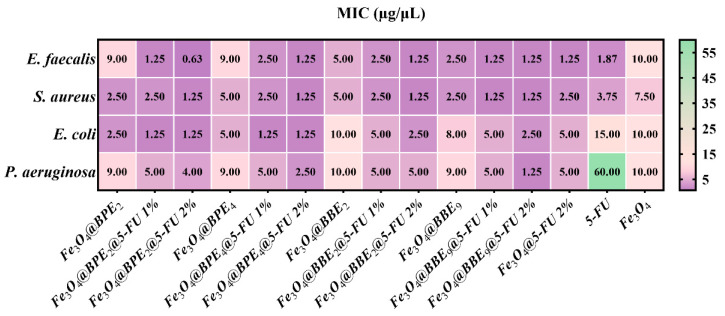
Heat map of MIC values. Data results marked in intense purple indicate the significantly lowest MIC values and highest sensitivity of strains. The scale bar shows the variations in the sensitivity of tested strains from the highest (purple) to the lowest (green).

Secondly, the qualitative antimicrobial assessment was continued using a qualitative assay by determining the MIC values (Figure 14), which are characterized by the smallest concentration of obtained MNPs that inhibit microbial growth. 

The MIC assay confirmed the qualitative data, and the most sensitive strains were represented by Gram-positive bacteria. In the case of Gram-negative bacteria, the MNPs loaded with BPEs determined the highest sensitivity for *E. coli*. In contrast, MNPs with BBEs presented moderate inhibition of Gram-negative bacteria, and *P. aeruginosa* was the most resistant. Additionally, for most of the MNPs, the MIC values were lower than Fe_3_O_4_@5-FU 2% and 2% 5-FU. The addition of extracts significantly inhibited the development of the strains, which can be explained by the antioxidant activity of the BPEs and BBEs, and by the PCs’ release behavior. 

The lowest MIC value was obtained for Fe_3_O_4_@BPE_2_@5-FU 2% (0.625 μg/mL) on *E. faecalis*. Also, the smallest MIC values were obtained for Fe_3_O_4_@BPE_4_@5-FU 2% and Fe_3_O_4_@BPE_9_@5-FU 2%, which range from 1.25 to 2.5 μg/mL for all strains. In contrast, the antitumoral drug determined less sensitivity of Gram-positive and Gram-negative bacteria than most MNPs. It is notable that at MIC of 0.625 μg/mL MNP, the contain of 5-FU is 0.012 μg/mL, which means a strong antibacterial activity of the drug when loaded on the MNP surface. The reduction of 5-FU dosage can possibly be improved due to the small dimensions of the synthesized MNPs (<10 nm), drug delivery efficiency (Figure 9), and antioxidant activity (Figure 10, Figure 11 and Figure 12) [103,104,105]. Likewise, according to the IC_50_ values (Figure 10), a smaller quantity of MNPs is necessary to scavenge the DPPH radical, corresponding to the greatest antioxidant activity and significant inhibitory effects. Furthermore, a recent paper confirms our results regarding the antibacterial activity of the drug, and reported that MIC values for 5-FU are 2 μg/mL for *S. aureus* ATCC 25923, 16 μg/mL for *E. coli*, and 256 μg/mL for *P. aeruginosa* [106].

Otherwise, Spulber et al. [107] reported that Fe_3_O_4_ functionalized with *p*-aminobenzoic acid and BPEs with irregular shapes and 40–60 nm sizes exhibited inhibitory effects on *S. aureus* ATCC 29213 (MIC: 120–500 μg/mL), *E. faecalis* ATCC 29212 (250–500 μg/mL), *E. coli* ATCC ATCC 25922 (120–500 μg/mL), and *P. aeruginosa* ATCC 27853 (250–500 μg/mL). From our knowledge, there are no references to compare our results regarding the positive impact of MNPs on the antibacterial properties of antineoplastic agents, especially the synergic effects between extracts, 5-FU, and MNPs. 

#### 3.4.3. Semiquantitative Assay of the Bacterial Adherence to the Inert Substratum

**Figure 15:**
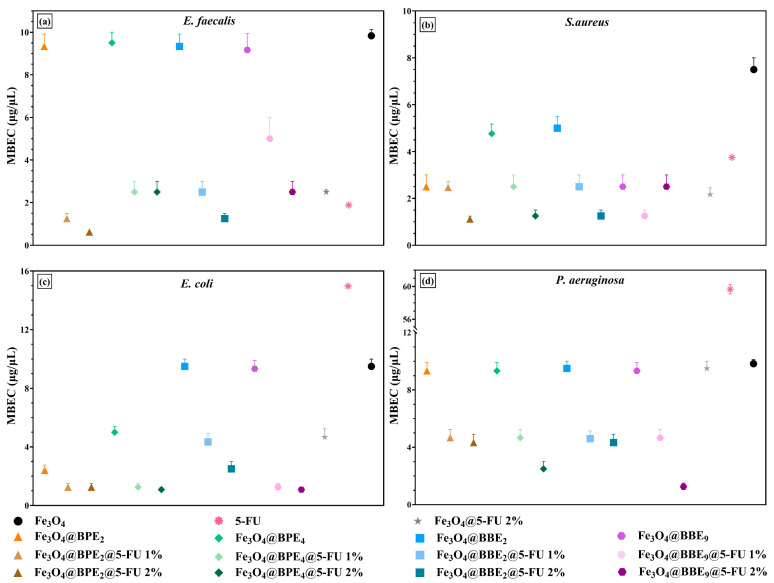
Graphical chart of MBEC values: (**a**) *E. faecalis*, (**b**) *S. aureus*, (**c**) *E. coli*, and (**d**) *P. aeruginosa*. The differences between MNPs were statistically analyzed using the one-way ANOVA followed by Tukey’s multiple comparisons tests. The data results were considered statistically significant.

Additionally, the influence of loaded MNPs on the pathogenic strains’ adherence to the inert substratum was assessed. The minimal biofilm eradication concentration (MBEC) values are graphically represented in Figure 15.

The antibiofilm data confirms the qualitative (Figure 13) and quantitative/MIC results (Figure 14). Therefore, *S. aureus* and *E. faecalis* were the most sensitive strains in the presence of developed MNPs. Likewise, loaded MNPs significantly inhibited the adherence of *E. coli* and had moderate activity on *P. aeruginosa*. 

The BPE-loaded MNPs presented similar antibacterial profiles, and the strongest antibiofilm activity was exhibited by MNPs loaded with BPE_4_. The antibacterial activity of MNPs loaded with BPEs could be explained by the higher phenolic and flavonoid content and the greatest antioxidant activity. Moreover, PCs show inhibitory effects and induce a disturbance in biofilm development and architecture [108,109]. Otherwise, the MNPs trigger oxidative stress and cell lysis with ROS production, affecting H⁺-fluxes through the bacterial membrane, DNA and ribosomal damage, inactivation of enzymes, and disrupting the cytoplasm and cell membrane [110,111,112]. Also, small sizes, long-term stability, and uniformity of the developed nanocarriers enable them to transport bioactive compounds to targeted tissues and penetrate the biofilms [113]. Regarding these MNPs’ properties, it could be explained as the improvement of 5-FU anti-adherence activity. However, according to our knowledge, there are no reference data to compare our results on the enhancement of 5-FU antibacterial activity when is loaded on the MNPs’ surface with BPEs or BBEs. 

### 3.5. Influence of Bioactive Compounds against Probiotic Bacteria

**Figure 16:**
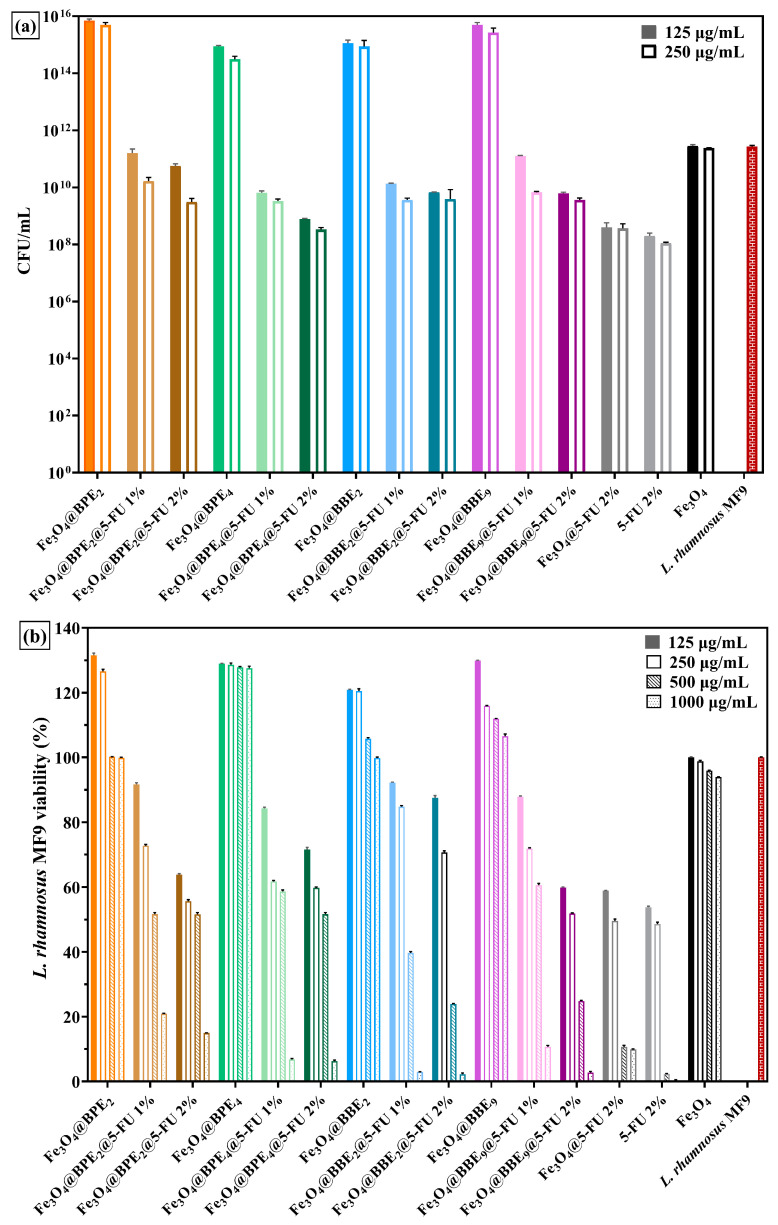
The influence of MNPs on the growth of *L. rhamnosus MF9* (after 24h). (**a**) Graphic representation of CFU/mL values. (**b**) A representation of the percentages of growth of probiotic bacteria viability under the influence of different concentrations of MNPs. The data results were considered statistically significant (*p* < 0.001).

The human gastrointestinal tract is characterized by complex microbial communities that generate many metabolites involved in numerous biological processes. The major challenge is to prevent dysbiosis that imbalances the REDOX equilibrium, which also damages the immune system by disrupting signaling and stimulating inflammatory responses [114]. Dietary antioxidants (PCs, fatty acids, vitamins, etc.) might prevent oxidative stress by regulating/restoring the gut microbiota [115,116]. The effect on the growth of *L. rhamnosus* MF9 under the influence of loaded MNPs after 24 h is represented in Figure 16. 

The Fe_3_O_4_ NPs do not influence the growth of *L. rhamnosus* MF9, but are significantly stimulated (more than four logarithmic units) by all BPEs and BBE-loaded MNPs (Figure 16a). Otherwise, Figure 16b illustrates the impact of different concentrations of MNPs against *L. rhamnosus* MF9 growing. The negative influence of the antitumoral drug can be observed at concentrations between 250 and 1000 μg/mL. Also, Figure 16a confirms the impact of 5-FU on the probiotic bacteria’ growth. At 125 μg/mL concentrations of MNPs, the *L. rhamnosus* MF9 growth was negatively influenced by the samples with 2% antineoplastic agent, less Fe_3_O_4_@BPE_2_@5-FU 2%. The significant prebiotic potential of this sample is correlated with the prebiotic activity of their extract (BPE_2_) and the antioxidant activity of loaded MNPs (Figure 10, Figure 11 and Figure 12). 

According to the CUPRAC and FRAP results (Figure 11 and Figure 12), the MNPs loaded with 5-FU 1% showed the highest antioxidant activity, and it could be correlated with the capacity of these samples to alleviate the oxidative stress induced by the anticancer drug against gut microbiota. Also, Figure 16a suggests that at 125 μg/mL MNPs, the growth of *L. rhamnosus* MF9 is not affected by the Fe_3_O_4_@BPE_2_@5-FU 1% and Fe_3_O_4_@BPE_9_@5-FU 1%. Previous studies [117,118] confirm the prebiotic effects of PCs on probiotic bacteria, and it could be assumed that bioactive compounds from extracts alleviate the adverse effects of 5-FU by protecting and enhancing the *L. rhamnosus* MF9 development. 

The non-toxic properties of MNPs for probiotic strains are confirmed by Ghibaudo et al. [119], who synthesized MNPs coated with citrus pectin used as carriers for *L. plantarum*. The iron from NPs was non-toxic up to 1 mg/mL. In addition, the prolonged PCs’ release profiles (Figure 9c,d) suggest an enhanced possibility of microbiota recovery. In addition, a high growth rate of probiotic bacteria does not affect the antitumoral effect of drugs [120]. 

### 3.6. The Antiproliferative Assay 

**Figure 17:**
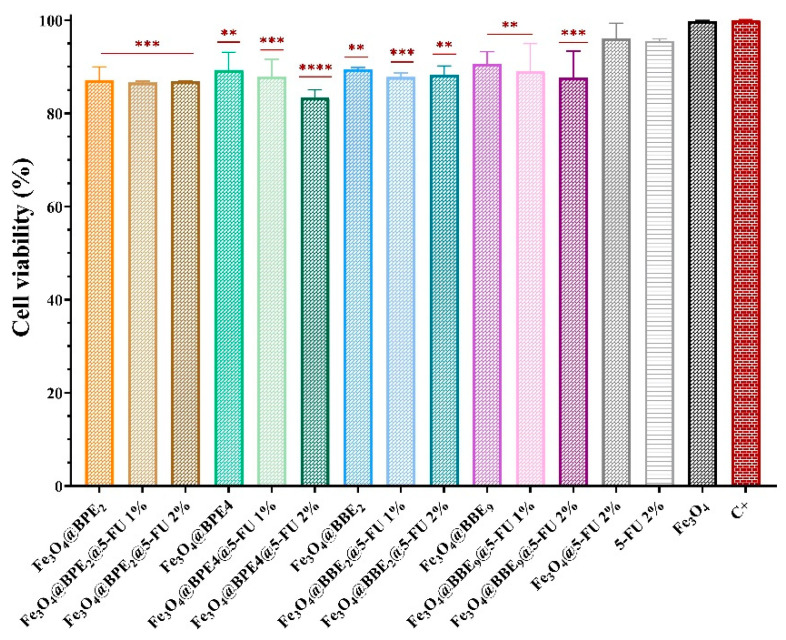
The influence of loaded-MNPs on the cell viability and proliferation of the colorectal tumoral cells (C+). The differences between the cell viability of HT-29 cells without MNPs (C+) and under MNPs’ influence for the MTT assay were statistically analyzed using one-way ANOVA followed by Holm–Šídák’s multiple comparisons tests (** *p* ≤ 0.005; *** *p* ≤ 0.001; **** *p* < 0.0001).

The in vitro tumor cell viability and proliferation of HT-29 cells in the presence of synthesized MNPs were examined using the MTT assay (Figure 17). The cell viability of the colorectal tumoral cells was assessed at the lowest concentration of MNPs (62.5 μg/mL), which does not inhibit the growth of probiotic bacteria or affect the gut balance. 

Figure 17 displays the significant differences between magnetite and loaded MNPs, and it reveals that the developed MNPs do not stimulate the proliferation of colorectal tumoral cells. 

Fe_3_O_4_@BPE@5-FU/Fe_3_O_4_@BBE@5-FU and Fe_3_O_4_@BPE/Fe_3_O_4_@BBE samples present a lower cell viability than Fe3O4@5-FU 2%. 

Likewise, adding 5-FU improves the inhibition of HT-29/tumoral cell proliferation, which could be explained by a synergic inhibitory effect between PCs from extracts and anticancer drug. Fe_3_O_4_@BPE_4_@5-FU 2% presented the lowest percentage of cell viability (83.3%) and the highest inhibitory effect, respectively. Also, Fe_3_O_4_@BPE_2_ with 1% and 2% 5-FU (~86.7%), Fe_3_O_4_@BBE_9_@5-FU 2% (87.6%), and Fe_3_O_4_@BBE_2_@5-FU 1% (87.8%) significantly inhibited the proliferation of tumoral cells. Otherwise, this study aimed to investigate the effects between 5-FU and extracts, especially the influence on gut microbiota, and for this reason it used small concentrations of 5-FU and MNPs and does not expect significant antitumoral activity. 

The antitumoral activity of PCs present in high amounts in the BP/BB extracts has already been studied [40,121]. Akkoyunlu et al. [122] accomplished the BP antitumoral activity on the HCT116 cell line, and Uțoiu et al. [123] reported a moderate inhibitory effect on Hep-2 cells and Caco-2 intestinal tumor cells of a fermented BP. In addition, the antitumoral activity of BB on CRC cell lines was reported [124]. 

The antitumoral properties of MNPs loaded with 5-FU are confirmed by another study [82], which reported that MNPs can play a role in improving 5-FU anticancer activity. Dextran-coated iron NPs loaded with 5-FU (1.5:1), with 30.19 ± 2 nm average particle size, also presented an antiproliferative effect on Caco-2 cells at 5–250 μg/mL NPs. Genc et al. [125] reported that spherical Fe_3_O_4_ NPs with 35 ± 5 nm average diameter do not inhibit the Caco-2-cells proliferation, but adding different concentrations of 5-FU enhanced the inhibitory effect. Moreover, the efficacy of loaded 5-FU on the chitosan-coated MNPs was improved by applying magnetic hyperthermia [126]. In our study, citrate-coated MNPs loaded with BPEs, BBEs, and/or lower/minor concentrations of 5-FU presented high stability, smaller particle sizes, lower IC50 values, and a high percentage of drugs released. Furthermore, the novelty of this study is represented by the development of the DDSs, which highlighted antibacterial, prebiotic, and antiproliferative activities.

Bacterial infections developed during the CRC treatment negatively impact the patient’s survival [127], and the antimicrobial action of new MNPs on pathogen strains can only be beneficial. The biological properties of the developed (magnetic) nanocarriers improve gut homeostasis and could ensure a targeted delivery at the colon site.

## 4. Conclusions

The present study aimed to develop drug delivery systems based on citrate-stabilized MNPs, which act as nanocarriers for PCs from extracts and anticancer drugs. The results demonstrated the achievement of MNPs with narrow size distributions and an average NP size of ~10 nm. Moreover, it was shown that the antitumoral drug and PCs had fast release rates in the first 24 h from MNPs. Furthermore, the PCs exhibited prolonged release behavior (up to 96 h), and loading MNPs with extracts enhanced their antioxidant and antibacterial properties. Additionally, the non-toxic properties of magnetite and the prebiotic potential of the developed DDS on *L. rhamnosus* MF9 were demonstrated, underline the role of the antioxidants (PCs) in supporting gut microbiota. The moderate antiproliferative activity on a colorectal tumoral cell line at low concentrations of MNPs and 5-FU suggests the potential of DDS to alleviate the adverse effects of anticancer drugs and improve gut balance. 

## Figures and Tables

**Table 1:** Release kinetics models applied.

Release Kinetic	Equation
Zero-order	$C_{t}=C_{0}+k_{0}\;\cdot t$
First-order kinetics	$\log C_{t}=\log C_{0}-k_{1}\cdot \frac{t}{2.303}$
Higuchi model	$C_{t}=k_{H}\;\cdot t^{1∕2}$
Hixson–Crowell model	$C_{0}^{1∕3}-C_{t}^{1∕3}=k_{HC}\cdot t$

*C_t_* is the amount of drug released in time (*t*); *C*_0_ is the initial amount of 5-FU in medium (usually is 0); *k*_0_, *k*_1_, *k_H_*, and *k_HC_* are the 5-FU release rate constants.

**Table 2:** Crystallite size for obtained MNPs.

Sample	Average Crystallite Size ± SD (nm)
Fe_3_O_4_	7.05 ± 1.09
Fe_3_O_4_@5-FU 2%	6.52 ± 0.77
Fe_3_O_4_@BPE_2_@5-FU 2%	7.29 ± 0.71
Fe_3_O_4_@BPE_4_@5-FU 2%	8.31 ± 2.49
Fe_3_O_4_@BBE_2_@5-FU 2%	6.19 ± 0.89
Fe_3_O_4_@BBE_9_@5-FU 2%	6.78 ± 1.30

**Table 3:** The thermal effects, mass loss, and estimated load.

Sample	1st Mass Loss % (Water Loss)	2nd Mass Loss% (Organic Part Oxidation)	Exo (°C) γ-Fe_2_O_3_ to α-Fe_2_O_3_	Residual Mass (%) at 900 °C	Estimated Load % (α-Fe_2_O_3_)
Fe_3_O_4_	1.87%	1.72%	586.3 °C	94.51%	-
Fe_3_O_4_@5-FU 2%	2.31%	3.40%	542.6 °C	92.63%	1.99%
Fe_3_O_4_@BPE_2_@5-FU 2%	1.75%	17.60%	565.0 °C	79.24%	16.16%
Fe_3_O_4_@BPE_4_@5-FU 2%	1.98%	16.65%	552.5 °C	79.80%	15.56%
Fe_3_O_4_@BBE_2_@5-FU 2%	3.55%	14.80%	550.7 °C	79.43%	15.96%
Fe_3_O_4_@BBE_9_@5-FU 2%	1.86%	5.47%	549.4 °C	91.27%	3.43%

**Table 4:** Release kinetic parameters.

**5-FU Release Kinetics**								
**Sample**	**Zero-Order Release Kinetics**		**First-Order Release Kinetics**		**Higuchi Release Kinetics**		**Hixson–Crowell Release Kinetics**	
	**K_0_**	**R^2^**	**K_1_**	**R^2^**	**K_H_**	**R^2^**	**K_HC_**	**R^2^**
Fe_3_O_4_@5-FU 2%	0.8241	0.8450	−0.0361	0.9225	5.2970	0.8670	−0.0323	0.9028
Fe_3_O_4_@BPE_2_@5-FU 1%	0.5260	0.8886	−0.0298	0.9357	3.2880	0.8755	−0.0241	0.9289
Fe_3_O_4_@BPE_2_@5-FU 2%	0.4837	0.8948	−0.0352	0.9521	2.9220	0.8315	−0.0269	0.9461
Fe_3_O_4_@BPE_4_@5-FU 1%	0.5648	0.8433	−0.0341	0.9331	3.6550	0.8958	−0.0270	0.9153
Fe_3_O_4_@BPE_4_@5-FU 2%	0.7443	0.9394	−0.0593	0.9888	4.7380	0.9177	−0.0436	0.9865
Fe_3_O_4_@BBE_2_@5-FU 1%	0.5922	0.7906	−0.0286	0.8699	3.8280	0.8201	−0.0235	0.8606
Fe_3_O_4_@BBE_2_@5-FU 2%	0.6153	0.5265	−0.0287	0.6962	4.2890	0.6641	−0.0235	0.6600
Fe_3_O_4_@BBE_9_@5-FU 1%	0.8436	0.7935	−0.0433	0.9203	5.5080	0.8494	−0.0364	0.8875
Fe_3_O_4_@BBE_9_@5-FU 2%	0.8094	0.7774	−0.0473	0.9213	5.2820	0.8436	−0.0412	0.8853
**PCs Release Kinetics**								
**Sample**	**Zero-Order Release Kinetics**		**First-Order Release Kinetics**		**Higuchi Release Kinetics**		**Hixson–Crowell Release Kinetics**	
	**K_0_**	**R^2^**	**K_1_**	**R^2^**	**K_H_**	**R^2^**	**K_HC_**	**R^2^**
Fe_3_O_4_@BPE_2_	0.9470	0.9001	−0.0460	0.8509	119270	0.9790	−0.0395	0.8891
Fe_3_O_4_@BPE_2_@5-FU 1%	0.6197	0.9482	−0.0186	0.9902	6.8219	0.9905	−0.0197	0.9786
Fe_3_O_4_@BPE_2_@5-FU 2%	1.0660	0.8569	−0.0669	0.9858	11.4270	0.9877	−0.0497	0.9743
Fe_3_O_4_@BPE_4_	0.6904	0.8967	−0.0430	0.8603	7.1410	0.9104	−0.0342	0.8799
Fe_3_O_4_@BPE_4_@5-FU 1%	0.5092	0.9484	−0.0413	0.9545	11.6673	0.9636	−0.0299	0.9625
Fe_3_O_4_@BPE_4_@5-FU 2%	0.7391	0.8316	−0.0332	0.6993	10.1680	0.9676	−0.0298	0.7750
Fe_3_O_4_@BBE_2_	0.8498	0.5559	−0.0400	0.8894	9.6730	0.6837	−0.0347	0.7869
Fe_3_O_4_@BBE_2_@5-FU 1%	0.7960	0.4252	−0.0464	0.7968	12.8670	0.5766	−0.0363	0.6749
Fe_3_O_4_@BBE_2_@5-FU 2%	0.9048	0.6576	−0.0578	0.9191	10.0700	0.7736	−0.0433	0.8800
Fe_3_O_4_@BBE_9_	0.9786	0.7716	−0.0444	0.9570	10.5900	0.8579	−0.0389	0.9252
Fe_3_O_4_@BBE_9_@5-FU 1%	0.8135	0.6334	−0.0266	0.8646	9.0240	0.7399	−0.0270	0.8075
Fe_3_O_4_@BBE_9_@5-FU 2%	1.2150	0.8028	−0.0552	0.9942	13.2500	0.9051	−0.0469	0.9609

## Data Availability

Data are contained within the article.
